# Long Continued Hemorrhage in the Latter Half of Pregnancy

**Published:** 1893-11

**Authors:** 


LONG-CONTINUED HEMORRHAGE IN THE LATTER HALF OF PREGNANCY, DUE TO DETACHMENT OF A NORMALLY-SITUATED PLACENTA, AND ACCOMPANIED BY SEPTIC INTOXICATION, WITH A REPORT OF TWO CASES.—Hirst (*Medical News*, July 22, 1893) reports two cases of hemorrhage from detachment of a normally-situated plancenta, both occurring at about the sixth month, and evidencing the absorption of septic products from the slowly-decomposing clots which had formed between the layers of the decidua vera.

The first case had been bleeding profusely from the genital passage for two or three weeks. She was profoundly reduced from loss of blood and had considerable fever. The child was still alive. Diagnosing premature detachment of the placenta, in view of the alarming condition of the woman, Hirst determined to terminate the pregnancy. The placenta was found to be attached at the fundus, with one-quarter of its maternal surface covered with blood-clot dark in color and firm as liver in consistence. Clots extended along the membranes to and beyond their point of rupture. The patient recovered after exhibiting symptoms of septicemia for a time.

The second case had been bleeding for six weeks, despite careful rest in bed, with opium and viburnum during the last four weeks. She had a septic temperature during this time.

Although the fetus was still alive it was determined to terminate the pregnancy. The placenta was found attached at the fundus. About one-eighth of its surface was degenerated from previous separation. A long, decomposing clot was attached to this margin. The woman made a good recovery.

These are of especial interest inasmuch as the text-books in common use do not describe this condition, and the general practitioner, unless he had seen and recognized such an accident, would be at a loss in handling his case.—*Ex.*

